# Considerations for Improved Mobile Health Evaluation: Retrospective Qualitative Investigation

**DOI:** 10.2196/12424

**Published:** 2020-01-22

**Authors:** Samantha Dick, Yvonne O'Connor, Matthew J Thompson, John O'Donoghue, Victoria Hardy, Tsung-Shu Joseph Wu, Timothy O'Sullivan, Griphin Baxter Chirambo, Ciara Heavin

**Affiliations:** 1 Health Information Systems Research Centre Cork University Business School Cork Ireland; 2 Department of Family Medicine University of Washington Seattle, WA United States; 3 Global eHealth Unit Department of Primary Care and Public Health Imperial College London London United Kingdom; 4 Research Department Luke International Mzuzu Malawi; 5 Faculty of Health Sciences Mzuzu University Luwinga, Mzuzu Malawi

**Keywords:** telemedicine, mHealth, research design, developing countries

## Abstract

**Background:**

Mobile phone use and, consequently, mobile health (mHealth) interventions have seen an exponential increase in the last decade. There is an excess of 318,000 health-related apps available free of cost for consumers to download. However, many of these interventions are not evaluated and are lacking appropriate regulations. Randomized controlled trials are often considered the gold standard study design in determining the effectiveness of interventions, but recent literature has identified limitations in the methodology when used to evaluate mHealth.

**Objective:**

The objective of this study was to investigate the system developers’ experiences of evaluating mHealth interventions in the context of a developing country.

**Methods:**

We employed a qualitative exploratory approach, conducting semistructured interviews with multidisciplinary members of an mHealth project consortium. A conventional content analysis approach was used to allow codes and themes to be identified directly from the data.

**Results:**

The findings from this study identified the system developers’ perceptions of mHealth evaluation, providing an insight into the requirements of an effective mHealth evaluation. This study identified social and technical factors which should be taken into account when evaluating an mHealth intervention.

**Conclusions:**

Contextual issues represented one of the most recurrent challenges of mHealth evaluation in the context of a developing country, highlighting the importance of a mixed method evaluation. There is a myriad of social, technical, and regulatory variables, which may impact the effectiveness of an mHealth intervention. Failure to account for these variables in an evaluation may limit the ability of the intervention to achieve long-term implementation and scale.

## Introduction

### Background

Mobile health (mHealth) is the use of mobile technologies to improve health care and public health [1]. The driving forces for mHealth are the clinician’s need for providing care at any time, in any place, and the rapid advancement of new and emerging mobile technologies [2]. The developing world has the fastest growing mobile phone subscriber market in the world [3], producing millions of potential points of care [4]. As a result, the use of mHealth interventions has increased [5]. However, there is little existing quality control, regulatory oversight, or understanding of the clinical utility or clinical impact of many of these apps. Research is needed to assess when, where, and for whom mHealth is beneficial [6]. Rigorous evaluation of these platforms is essential for estimating their impact, along with the potential risks and benefits for end users, consumers, and the health care system as a whole [7].

### The Evaluation of Mobile Health

The current evidence for the efficacy of mHealth interventions is sparse [3,6,8-11], which may be because of a lack of high-quality, rigorous evaluations [12], with many mHealth projects explored only at the pilot phase [13]. In addition, there is limited information on the resources that should be invested in evaluation, and mHealth developers are citing the need for greater support and guidance when evaluating their projects [12]. Currently, there is little consensus on the methodological standards for evaluating mHealth interventions [4,14,15], but calls for more rigor in evaluation have led to an increase in the number of mHealth randomized controlled trials (RCTs) conducted in developed and developing countries [8,16,17].

RCTs are typically considered to be the *gold standard* study design for determining the effectiveness of clinical interventions [18] and are commonly used for mHealth evaluations [19]. However, there are increasing suggestions that RCTs may be impractical for mHealth evaluation [20,21]. mHealth interventions are inherently challenging to evaluate because of the fast moving and evolving technologies resulting in many platforms becoming obsolete even over the course of a single clinical trial; the high level of financial, human, and time resources needed to conduct rigorous evaluations; the complexity of many mHealth interventions, with regard to outcome measures of the intervention itself; the involvement of a multidisciplinary team; and the complex sociotechnical aspects on which the success of mHealth depends [10,19,22]. These factors make it difficult to adhere stringently to the standards and practicality of conducting RCTs for mHealth and using them to inform practice and policy decisions. The lack of a unified or standardized approach to mHealth evaluation is a major weakness and threatens the credibility of mHealth [9] as a premature scale-up of an mHealth initiative could harm the entire field [10,23].

### Objective of the Study

The aim of this qualitative study was to explore mHealth evaluation, identifying the factors contributing to an effective evaluation. We used the context of an ongoing mHealth project to explore the perspective of system developers directly involved in designing and evaluating an mHealth solution.

## Methods

### Overview

A qualitative approach was employed to facilitate deeper exploration of the factors that were instrumental in deciding how to evaluate the mHealth solution [24]. This study gathered data from a multidisciplinary sample of system developers (combining technical, clinical, managerial, and operational personnel) working on a single mHealth-based trial, incorporating those responsible for building the mHealth system, including software developers, health care professionals, and researchers [25]. This study was conducted as part of the first author’s master’s degree research.

### Study Setting—Randomized Controlled Trial for a Mobile Health Intervention in a Developing Country (The Supporting Low-Cost Intervention for Disease Control Project)

The Supporting Low-cost Intervention For disEase control (Supporting LIFE) project was a European Commission–funded project aimed at addressing child mortality rates in the under-5 population in Malawi, Africa [26]. As malaria and infantile diarrhea are the 2 main causes of mortality in this area, an mHealth project was designed to provide low-cost, effective, and targeted intervention in remote and resource-poor settings to overcome inadequate health care infrastructures. The project included a multinational group of experts, institutions, and nongovernmental organizations in the United Kingdom, Ireland, Sweden, United States, Malawi, and Switzerland. The project supported health surveillance assistants (Malawian term for community health workers) at the point of patient care to aid the community health service delivery to children under 5 years. It utilized mobile technology, existing application programming interfaces, and a clinical decision support system to support the limited health care infrastructure. The mHealth intervention was an Android-based smartphone app developed by the project consortium for use by health surveillance assistants in rural communities. The services provided by the health surveillance assistants followed the integrated Community Case Management of the Ministry of Health, adopted from the World Health Organization and the United Nations Children’s Fund guidelines. The app replicated the validated paper-based integrated Community Case Management guidelines from the Ministry of Health, with decision aid and logic checks to be used by health surveillance assistants in routine practice in Malawi. The mobile app was evaluated in a pragmatic, stepped-wedge cluster RCT between October 2016 and February 2017. The trial recruited 102 health surveillance assistants and 6995 patients.

### Participants and Recruitment

Recruitment for this investigation took place within the context of the Supporting LIFE project being conducted in Malawi [26]. Participants were selected using positional and reputational methods, techniques that have been developed to identify key participants for research [27]. Positional methods involved identifying persons who occupy key roles in a system [27]. In the case of an RCT, these individuals include the principal investigator and the project coordinator. Reputational methods involve identifying individuals believed to have the power to *move and shake* the system [27]. In the case of an RCT, these individuals include the project manager and the trial manager. Participants comprised a multidisciplinary group of system developers, encompassing all aspects of clinical trial and mHealth experience across a spectrum of clinical, technical, managerial, and operational disciplines. This cohort of participants was identified as being able to provide rich insights into the diverse aspects of an mHealth evaluation. A total of 15 system developers were identified from the project consortium. Table 1 outlines the project role and background of the participants in each category.

For inclusion in this study, participants were required to be currently or previously involved in an mHealth evaluation, aged 18 years or above, and fluent in spoken English. Participants were contacted by email in December 2017 to invite them to partake in the study. No individuals declined participation.

**Table 1:** System developers’ project roles and backgrounds.

System developers’ category	Participant identifier	Project role	Participant background
Clinical (n=4)	C1, C2, C3, and C4	Advisory committee Ethical application Clinical partner Data monitoring committee	Primary care and family medicine Infectious diseases
Technical (n=5)	T1, T2, T3, T4, and T5	Surveillance Testing Engineering lead Team leader App update	Information systems Decision support systems System architecture design Disease surveillance Software development
Managerial (n=2)	M1 and M2	Lead investigator Principal investigator	Health information systems Global health and electronic health Computer science
Operational (n=4)	O1, O2, O3, and O4	Scientific activity monitoring Project support Investigator Trial manager	Electronic health Global health Mental health Noncommunicable diseases

### Data Collection and Analysis

An interview guide was developed for the purpose of this study, and semistructured interviews were used to collect data from the participants in Malawi in January 2017. All potential participants were contacted before the interview to request their permission to participate in the study. All participants were provided with information sheets outlining the purpose of the research and consent forms, which they signed and returned by hand or by email before the interview. A conventional content analysis approach was used to analyze the transcripts [28,29]. All interviews and data analysis were conducted by 1 researcher (first author). A total of 9 private face-to-face interviews were conducted on the ground during a week-long field trip to Malawi in January 2017. Furthermore, 1 face-to-face and 5 Skype interviews were conducted with participants who were not available in Malawi. All interviews were audiorecorded and transcripts were returned to the participants on request.

The 15 interviews were transcribed verbatim. Before beginning coding, the interview audio was played alongside the transcript to allow for refamiliarization with the data and identification of any transcription errors. Line-by-line open coding was carried out by hand for 3 manuscripts. Accumulated codes were entered into NVivo 11 software (QSR) to allow for the organization and management of codes. Several codes were renamed or merged at this stage. Hand coding continued with each transcript and subsequent entry of codes into NVivo 11. Following the completion of open coding, 167 codes were identified. A visual mapping exercise was conducted to identify similar and duplicate codes and to group codes into categories. After the merging of similar codes and the removal of redundant codes, 4 major themes were abstracted from the categories [24]. A sample of the coding process is presented in Multimedia Appendix 1.

### Ethical Considerations

Ethical approval for this study was granted from the Social Research Ethics Committee at University College Cork. All data were anonymized at source, and participants are represented by their role in the study. The reporting of this study adheres to the consolidated criteria for reporting qualitative research guidelines [30] (see Multimedia Appendix 2).

## Results

### Summary of Results

In-depth interviews were conducted with 4 clinical, 4 operational, 5 technical, and 2 managerial team members of the Supporting LIFE project. Participants collectively contributed 425 min of interview time. Participants were predominantly males (n=11), with a mean age of 42 years (range 27-66 years). Most participants held a PhD (n=9), and over half of the participants (n=8) had prior experience with at least one mHealth evaluation. A total of 4 major themes emerged during the discussions of mHealth evaluation: (1) developing world context, (2) end users’ experience, (3) challenges to mHealth evaluation, and (4) mHealth regulation. Table 2 presents an illustration of the number of references to each theme by each project role category.

For clinical participants, the predominant focus was on mHealth challenges, followed by the regulatory issues in mHealth. Operational participants focused on mHealth challenges, the developing country context, and end users, with very little focus on mHealth regulation. Both technical and managerial participants were predominantly concerned with both end users and mHealth challenges.

**Table 2:** Number of theme references by project role category.

System developers’ category	Context	End users	mHealth challenges	Regulation
Clinical	27	25	63	34
Operational	15	13	16	1
Technical	13	56	45	18
Managerial	7	23	27	18

### The Developing World Context

The developing world context incorporated 3 subthemes: (1) infrastructural limitations, (2) perceptions of mobile phones, and (3) end users’ technological ability. All participants (n=15) discussed the impact of context on the evaluation of mHealth and the particular challenges of a developing country context:

Contexts are vastly different from one country, and sometimes even one area in a country to another.

C1

#### Infrastructural Limitations

A predominant focus was on the infrastructural limitations mentioned by most participants (n=11). One example was the issue of inadequate health record data; in Malawi, there are missing and incomplete birth and death registries as well as severely inadequate health records. Participants spoke of these issues being “out of our control” (O3) and having to “go with practicalities” (O2). Decisions were “heavily dependent on the infrastructure” (C3) to facilitate them:

Telecommunications was a big factor for us, the lack of network connectivity.

T5

#### Perceptions of Mobile Phones

A number of participants (n=5) discussed concerns regarding potential negative impacts of end users in varying contexts, namely, the health surveillance assistants. Potential “unhappiness” (C2 and C3) concerning the random allocation of smartphones could influence trial design changes, introduce biases, and jeopardize the success of the trial. It was suggested that this may be a problem in developing countries as “not everyone has a mobile device” (T5), and these devices are often perceived as being “valuable” and “exciting” (C2):

People without the device in the control group may get unhappy and withdraw.

C2

These interventions carry a lot of prestige, and they’re automatically seen as better and more reliable, and patients view health workers with these gadgets differently.

C3

#### End Users’ Technological Ability

Furthermore, the differences between the abilities of the technology developers and the end users were highlighted as a potential challenge as the gap is likely to be more pronounced in developing countries. Technology developers are “tech-savvy” (T2 and C1) and have a deep understanding of the characteristics of technology, but the end user, particularly a user in a low- or middle-income country, may have had very limited exposure to technology and may struggle with carrying out simple commands:

We’re developing technologies in a different context, we can’t expect that they’re just going to run the same way they would here... we need to get on the ground and talk to people, and really understand the cultural barriers and the cultural opportunities associated with using these technologies.

M2

### The End User’s Experience

The end user’s experience incorporated 2 subthemes: (1) understanding the end user and (2) the need for qualitative data. A deep understanding of the end users of the mHealth intervention was highlighted as key by all participants (n=15). Several participants (n=7) emphasized the importance of the end users’ involvement throughout the development and evaluation of the intervention:

You do want to know what user perceptions of the device are because uptake and successful long-term adoption is dependent on acceptability of the end users themselves.

C3

If the stakeholders aren’t happy with it, it’s never going to take off.

C3

#### Understanding the End User

Over half of the participants (n=8) discussed the importance of understanding the user experience of the mHealth intervention. Aspects of user experience included the user’s understanding and knowledge of the intervention (n=4) and the user’s interaction with the intervention (n=4). It was suggested that if the end users are not aware of the contribution they are making by using the mHealth tool, their decision to adopt the mHealth intervention in the long term could be adversely affected:

Do they [the Health Surveillance Assistants] fully appreciate the affordances of being able to contribute to that dataset… and the potential or advantages and derived value for public health and policy?

M2

Furthermore, several participants (n=6) discussed the importance of producing an mHealth intervention that does not place a burden on the end user. An mHealth intervention that fails in this area is more likely to fail in the long-term implementation:

[We should be designing] a technology that does its job, does it really well but in a really inconspicuous way so that the person can get on with doing all of the other really important things that they do.

M2

How comfortable or convenient is it for the person to use?

T2

#### The Need for Qualitative Data

The benefits of qualitative data were frequently mentioned by almost all participants (n=14), in terms of contributing to a deep understanding of the end users’ experience, suggesting its immense importance in mHealth evaluation. The rich understanding of the end users required for successful mHealth adoption cannot be achieved without the collection and analysis of qualitative data:

If we’d have not measured these qualitative elements, we would have missed many important benefits.

C2

We took the decision that in order to really understand the challenges around using and adopting the technology that we needed to use interview, focus group type techniques to actually explore the rich data around that.

M2

The interface with the community… going deep into where they are in their natural environment... you get very important information.

O1

### Challenges to Mobile Health Evaluation

The challenges of mHealth evaluation incorporated 3 subthemes: (1) mHealth complexity, (2) external influences, and (3) multidisciplinary involvement. The challenges of mHealth evaluation were discussed by all participants (n=15).

#### Mobile Health Complexity

The complexity of mHealth interventions was frequently mentioned by several participants (n=6), with particular focus on identifying a primary outcome measure for this mHealth study. It was also highlighted how this problem is compounded by the vast spectrum of mHealth apps and their varying complexity:

mHealth interventions are not black and white, there are so many aspects that you need to measure… how do you synthesise that into one trial because you have a limited number of outcomes because you can measure enough but you get to the point where it’s just making it really complicated, there are so many different outcomes that we’re measuring and I think that is a challenge.

C3

The RCT is the gold standard and if you get an RCT that is showing you a good positive result then you know, thumbs up, everyone is happy about that, but if it shows a negative result, you know, that sort of kills your project in a sense so it could have sort of, an unintended negative consequence in that it writes off your intervention as being useless when actually it might not be useless, it might actually be quite useful, it’s just you just didn’t measure the right outcome measure.

C1

For mHealth, I think there are so many other variables that it makes it much more difficult.

M2

#### External Influences

Almost all participants (n=13) discussed the external influencers of the evaluation design. For example, high-level stakeholders such as the Ministry of Health influence the type of evaluation used. These key decision makers often control the ongoing financial support for the interventions and their long-term implementation. Other participants spoke of the importance of having government-level stakeholders involved to ensure financial and political support after the initial research funding comes to an end:

I think by putting the RCT as a prerequisite up front it might help you to secure research funding.

T5

Malawi’s Ministry of Health are actively encouraging as many rigorous trials on mHealth technologies as possible, but they also want to gain an understanding of why they are potentially beneficial... I think that contributed to our decision to include a qualitative component.

C3

In terms of protocols and monitoring and the ethical side of things, it’s something we know how to do and I think research institutions in general are relatively comfortable with the idea of a RCT.

M2

It’s important because it is an international project… for the credibility of the whole research and the institutions.

T2

Furthermore, participants mentioned other influences as the outcome measures (n=5) and the availability of resources (n=4):

It depends on what you are measuring, so if you’re measuring just truly clinical outcomes, I suppose it doesn’t necessarily capture the technical issues.

C3

This trial specifically is a stepped-wedge approach and that was changed a few months before we actually implemented the study… it was resource constrained.

O3

#### Multidisciplinary Involvement

Participants from all 4 role categories (n=7) spoke about the challenges involved with the evaluation of an mHealth intervention, which requires the involvement of a multidisciplinary group of individuals, often from different institutions in different countries. Although all project members spoke English, overcoming disciplinary differences to find a *common language* among the members of an mHealth project proved challenging:

One of the key barriers to evaluating mHealth interventions is you have all these people coming together from different disciplines and none of them speak the same language.

C3

Although challenging, participants acknowledged the benefits of the diverse skill set. One-third of the participants (n=5) identified the general lack of evaluation in the field as a limitation in the guidance for conducting future mHealth evaluations. Most participants (n=12) identified the need for an alternative evaluation.

### Mobile Health Regulation

The mHealth regulations incorporated 2 subthemes: (1) lack of standards and (2) development of a hierarchy of risk. Two-thirds of the participants (n=10) discussed the regulatory issues in mHealth.

#### Lack of Standards

The most commonly raised issue was the lack of minimum standards (n=8) in the present mHealth evaluations globally. Several issues with setting a minimum standard were identified. First, the sheer volume of mHealth apps currently available is too great to suggest that RCTs should be conducted for each; hundreds of thousands of apps “are not going to have trials done” (C2). Second, the difficulty of deciding which type of evaluation should be conducted was emphasized. It was suggested that the type of evaluation should depend on the type of mHealth being evaluated, such as an app providing information or testing or diagnosis, and perhaps that aspect should inform the standards for mHealth evaluation:

When they start moving away from consumer health devices, to more medical devices needing some regulatory approval or evidence or proof for a country to adopt them or pay for them... what is that bar?

C2

When you read the guidelines, they’re a bit ambiguous and I think that it would really help my perception of when a RCT should be used.

T5

In addition, participants questioned the level of evidence required and whether an RCT was truly needed. The absence of standards for mHealth evaluations are potentially impacted by the lack of a clear definition of what exactly constitutes an mHealth intervention:

I think you’ve got to weigh up the benefits of going to the rigour of an RCT and the necessary requirements... versus whether [the intervention] could be evaluated by something simpler such as a before and after.

C4

Whether you call them mHealth or not depends on the definition.

C2

#### Development of a Hierarchy of Risk

Tying in closely with minimum standards is the development of a hierarchy of risk. This would allow for the classification of mHealth interventions based on their level of risk. mHealth is a broad term encompassing varying types of intervention, with differing levels of risk associated with each type. Several participants (n=6) spoke of the risk or level of anticipated harm and how it would contribute to defining standards and regulations and also how it could determine the type of evaluation design required for a particular mHealth intervention. A particular challenge across this theme was highlighted by several participants (C2, M1, and T2): “Who is going to take responsibility for it?” Questions were asked as to whether it should be an industry or governmental problem, if app stores should take the responsibility, or if there should be national and international policies in place.

## Discussion

### Principal Findings

This study aimed to explore the system developers’ experiences of mHealth evaluation to identify factors contributing to an effective evaluation. This study was conducted within the context of an ongoing cluster randomized clinical trial of an mHealth intervention being conducted in Malawi. Participants identified the impact of the developing country context. These include deficiencies in the existing health data systems; poor infrastructure such as roads, buildings, and telecommunications affecting data transfer and storage; and differing perceptions of mobile phone value, particularly smartphones, among study participants, impacting their involvement in the study. Emphasis was placed on the need to gain a comprehensive understanding of the end user’s experience of the intervention, and the importance of qualitative data collection and analysis was frequently mentioned. To ensure that the mHealth intervention being designed and developed is usable and useful, we need rich data to understand the end user’s needs, experiences, and attitudes toward the intervention and its potential deployment. This would promote the adoption of mHealth intervention and is a positive step toward enhancing the possibility of implementation in the future [31].

Several challenges were highlighted that potentially impact on the type of evaluation chosen for mHealth interventions. These included the complex nature of mHealth interventions; selecting appropriate outcome measures; the influence of funders, regulatory agencies, and multidisciplinary project teams; and an overall lack of evaluation across the field of mHealth, which limits the guidance available to project teams. Participants further identified regulatory issues in the field of mHealth, namely, the lack of minimum standards to guide evaluation. Participants discussed the benefits of devising a hierarchy of risk to inform mHealth evaluation.

### Comparison With the Literature

Technology and the people who use it are interdependent, each affecting the other [32]. The successful adoption of mHealth depends on the ability of the end user to operate the device and understand the technology. In a developing world context in particular, it is likely that the design-actuality gap [33] is large, so it is imperative that a comprehensive understanding of the social factors influencing mHealth is sought. The social aspects of mHealth include the social, cultural, religious, and behavioral interactions of the end user [10]. The importance of the end user’s involvement in the mHealth project from the outset was highlighted. Qualitative data collection and analysis is essential to derive rich insights from the end users. Utilizing qualitative data allows for the determination of social and contextual issues, desired effects, and usage factors [34,35]. The findings outline the aspects of the end user’s involvement that are critical to the long-term success of an mHealth intervention. The significance of the inclusion of qualitative evaluation is clear; this was highlighted in the Supporting LIFE project where a qualitative approach was embedded within the RCT, but this raises questions about current evaluations that fail to account for the unique characteristics of the mHealth apps they are evaluating [19].

The lack of regulation in the area of mHealth as outlined by Boudreaux et al [14] is supported by these findings. The potential damage to the credibility of the field of mHealth was highlighted by several participants who admitted the ease with which an unregulated, untested app could be released for public use. This finding has ramifications for mHealth as an area of study, and action must be taken to protect the patients and consumers of these apps, researchers, funders, and the reputation of mHealth. However, this study uncovered a challenge to the development of standards, which is compounded by the complexity of mHealth and the differing levels of risk involved within the diverse spectrum of available mHealth interventions. These complications may stem from the definition of mHealth, which encapsulates many technologies from sophisticated mobile medical devices for specific diseases and treatments to free apps for public use. The broad nature of the definition creates ambiguity when attempting to define standards by which mHealth interventions should be measured. This study emphasizes a number of challenges to the evaluation of mHealth, in support of the existing literature [6,7,19,22,36], highlighting an opportunity for the development of new methods for evaluating mHealth, which are able to adequately evaluate the complexities of mHealth interventions.

### Implications

To the best of our knowledge, this is one of the first studies to conduct an in-depth exploration of mHealth evaluation in the context of an ongoing clinical trial, and it contributes an urgently needed evidence base on the unique challenges of mHealth evaluation. Qualitative data can uncover important differences in the study populations, such as why a technology may work in one area but not in another, uncovering cultural, age, and education-related issues which quantitative data would fail to identify, and this is a major weakness in the use of an RCT alone for mHealth evaluation. In addition, the technical aspects identified are particularly important in the developing country context as mobile phone usage is vastly different, both in terms of the quality of the device and user ability. The findings from this study could contribute to the development of a more suitable, highly rigorous, cost-effective, and timely evaluation technique for mHealth. In the absence of clear consensus on mHealth evaluation, an appropriate next step may be the development of a decision support tool to enable mHealth project teams to identify the optimum study design or designs to select for evaluation using objective criteria, which could include quantitative, qualitative, or mixed method designs of various types.

mHealth incorporates a variety of interventions, with varying levels of risk associated with each. Therefore, a one-size-fits-all evaluation approach is unlikely to be suitable for mHealth, despite the external influence of funders and institutions. An mHealth intervention can be assessed from multiple perspectives, depending on the goals of the stakeholder. However, there should, at best, be a minimum standard of evaluation depending on the type of mHealth intervention. All mHealth interventions should pass a minimum standardized certification as to their quality, but mHealth interventions which aim to have a quantifiable impact on health should be further subject to a rigorous evaluation. One potential solution to the regulatory problems highlighted in this study is the development of a hierarchy of risk. If an intervention has a low risk of anticipated harm, such as an app giving clinical information, then a less rigorous evaluation design would be suitable, as opposed to an app that is more complex, requiring data from multiple sources, such as a brand-new decision support tool. Classifying mHealth as low-, medium-, and high-risk interventions would be based on factors such as the novelty of the intervention and the level at which it intervenes (and thereby potential risk) with human health and well-being. For example, interventions that provide descriptive information could be categorized as low risk; medium-risk interventions could include calorie and exercise tracking; and high-risk interventions could include diagnostic and treatment-centric interventions, which provide a prescriptive element.

White et al [21] outline that a successful mHealth evaluation should examine user feedback and outcome measures as well as the robustness of the technology, intervention principles, engagement strategies, and user interaction. Several alternative evaluation techniques to the RCT have been proposed, for example, continuous evaluation of evolving behavioral intervention technologies (CEEBIT) [37], the multiphase optimization strategy (MOST) [38], the sequential multiple assignment randomized trial (SMART) [39], and the microrandomized trial [40]. The next steps are required to determine the minimum level of evaluation and regulation required at each risk level. Using a hierarchy of risk as a guideline, mHealth project teams could justify their evaluation technique based on the evaluation requirement, perhaps avoiding situations where the evaluation technique is used to justify the funding. This will be particularly important as mHealth is adopted in developing countries where resources are scarce. On a larger scale, identifying an entity to take responsibility for the regulation and minimum standards of mHealth as a whole is extremely challenging, given the large reach of the mHealth field and the involvement of multidisciplinary research teams, ministries of health, app stores, and private industry.

### Limitations

This study has a number of limitations. First, the sample size of this study is small as it included only 15 participants. However, determining an adequate sample size in qualitative research is ultimately a matter of judgment in evaluating the quality of the information collected [41]. Participants in this study were selected using positional and reputational methods [27] to identify the key actors in an mHealth evaluation, but all participants from this study were part of the same mHealth project and may not be representative of other mHealth projects, which may be conducted in different contexts. Future research should explore mHealth evaluations in different contexts to identify challenges and considerations for successful evaluation. The field study methodology pursued in this study allowed the research to be conducted in the natural setting of an ongoing mHealth evaluation in a developing country, producing a rich, detailed insight of the evaluation process. Finally, all interviews and data analysis were conducted by 1 researcher. This is a weakness as qualitative data analysis is subjective and open to interpretation, but this has been mitigated by using analyst triangulation [42], whereby several of the study participants reviewed, discussed, and refined the findings of this study. Furthermore, a sample of the inductive, open coding approach has been provided in Multimedia Appendix 1.

### Conclusions

Contextual issues represented one of the most important challenges to evaluating an mHealth intervention in a developing country context and highlighted qualitative evaluation as imperative to ensure that the sociotechnical needs of end users are considered. The failure of mHealth interventions to address social and technical problems could have a profoundly damaging effect on the chances of long-term implementation and must be identified early on. Although RCTs have several important limitations in the mHealth context, the use of this rigorous evaluation methodology is the best approach in the absence of appropriate alternatives. However, it should be acknowledged that new evaluation methodologies are emerging, such as the CEEBIT, MOST, and SMART methodologies, which may be more suited to the complexities of mHealth, and project teams should be open to exploring these alternatives. There is an opportunity to design alternative approaches to mHealth evaluation, incorporating the hierarchy of risk, which challenge the one-size-fits-all approach and provide greater guidance and flexibility in evaluating different mHealth interventions in different contexts.

## Appendix

Multimedia Appendix 1 Sample of coding process.

Multimedia Appendix 2 Consolidated criteria for reporting qualitative research checklist.

## Abbreviations

CEEBIT: continuous evaluation of evolving behavioral intervention technologies
mHealth: mobile health
MOST: multiphase optimization strategy
RCT: randomized controlled trial
SMART: sequential multiple assignment randomized trial
Supporting LIFE: Supporting Low-cost Intervention For disEase control
