# Supplementary material for: Considerations for Improved Mobile Health Evaluation: Retrospective Qualitative Investigation
Source: JMIR Mhealth Uhealth. 2020 Jan 22;8(1):e12424. doi: 10.2196/12424 (PMC7003121; doi:10.2196/12424)
Supplement: Multimedia Appendix 1 [file mhealth_v8i1e12424_app1.docx]

| **Text** | **Code** | **Emerging Theme** |
| --- | --- | --- |
| *“Uptake and successful long term adoption is dependent on acceptability of the end users themselves” – C3.* | **Understanding the end-user** | **End-User Experience** |
| *“If we’d have not measured these qualitative elements, we would have missed many important benefits” – C2.* | **Qualitative data collection** |  |
|  |  |  |
| *“mHealth interventions are not black and white, there are so many aspects that you need to measure” – C3.* | **mHealth Complexity** | **Challenges to mHealth Evaluation** |
| *“I think by putting the RCT as a pre-requisite up front it might help you to secure research funding” – T5.* | **External Influences** |  |
| *“One of the key barriers to evaluating mHealth interventions is you have all these people coming together from different disciplines and none of them speak the same language” - C3.* | **Multi-Disciplinary Involvement** |  |
|  |  |  |
| *“You don’t know whether the referral rate was too high or too low to start with” – C1.* | **Infrastructural Limitations** |  |
| *“Because were developing technologies in a different context, we can’t expect that they’re just going to run the same way they would here” – M2.* | **Technological Ability** | **Developing World Context** |
| *“In this setting, a mobile phone is such a valuable tool” – C2.* | **Perceptions of Mobile Phone** |  |
|  |  |  |
| *“When they start moving away from consumer health devices, to more medical devices needing some regulatory approval or evidence or proof for a country to adopt them or pay for them… what is that bar?” – C2.* | **Lack of Standards** | **mHealth Regulation** |
| *“I think you’ve got to weigh up the benefits of going to the rigour of an RCT and the necessary requirements… versus whether [the intervention] could be evaluated by something simpler such as a before and after” – C4.* | **Hierarchy of Risk** |  |

## **Appendix A** – Sample of coding process
